# Dispersion of arc-discharged single-walled carbon nanotubes using the natural α-amino acid derivative *N*-dodecanoyl leucinate†

**DOI:** 10.1039/d0ra02862b

**Published:** 2020-06-05

**Authors:** Heng Zhao, Lihua Guo, Yongfu Lian

**Affiliations:** Key Laboratory of Functional Inorganic Material Chemistry, Ministry of Education, School of Chemistry and Materials Science, Heilongjiang University Harbin 150080 China chyflian@hlju.edu.cn +86 451 86608576 +86 451 86608576; Key Laboratory of Photochemical Biomaterials and Energy Storage Material, School of Chemistry and Chemical Engineering, Harbin Normal University Harbin 150025 Heilongjiang China

## Abstract

The natural α-amino acid derivate *N*-dodecanoyl leucinate was synthesized *via* Schotten–Baumann reaction and alkali treatment, and was applied to the dispersion of arc-discharged single-walled carbon nanotubes (SWNTs). Optical absorption and Raman scattering spectra as well as AFM observation confirmed the effective individualization and selective dispersion of SWNTs. Moreover, charge transfer from *N*-dodecanoyl leucinate to SWNTs was evidenced by FT-IR and Raman scattering spectroscopic analyses. We believe that the formation of a charge transfer complex between dispersants and SWNTs is responsible for the effective individualization of SWNTs, and that the charge transfer from dispersants to SWNTs (or from SWNTs to dispersants) is crucial for selective dispersion of semiconducting (or metallic) SWNTs.

## Abstract 2

The synthesized natural α-amino acid derivative *N*-dodecanoyl leucinate demonstrates an effective and selective dispersion towards arc-discharged SWNTs.

## Graphical abstract

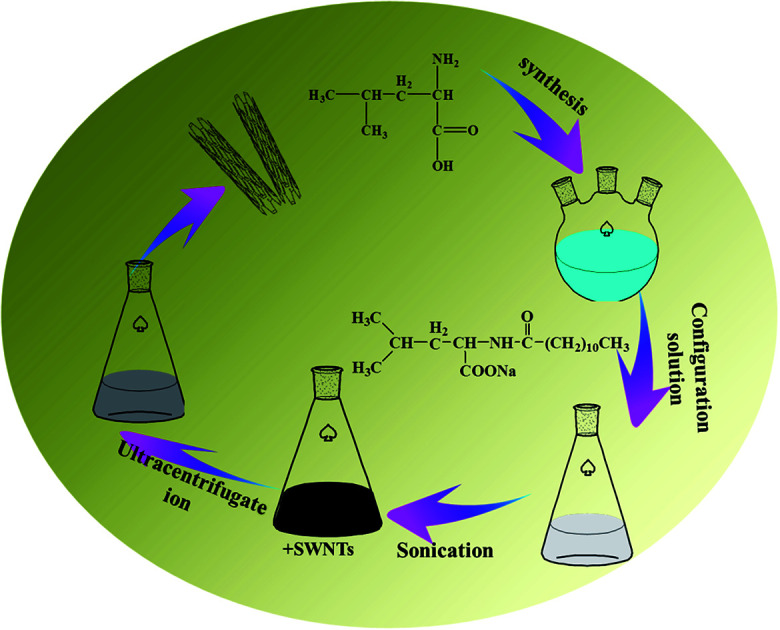

## 1 Introduction

Single-walled carbon nanotubes (SWNTs) have great potential because of their excellent electronic, thermal, optical, and mechanical properties.^1–3^ Particularly, individual SWNTs play very important roles in the fields of scanning probe microscope, drug delivery, chemical or biological sensing and miniaturization of electronic circuitry.^4–8^ Unfortunately, the available preparation techniques yield inevitably huge and close bundles of SWNTs, which imposes a considerable challenge for their separation and assembly in both aqueous and non-aqueous solution.^9^

To date, postsynthetic dispersion is the only practical approach to obtain individual SWNTs on a large scale, and SWNT bundles could be individualized by covalent or noncovalent chemical modification.^10,11^ In sharp contrast to covalent individualization, the noncovalent one generates individual SWNTs with preservation of their intrinsic electronic and mechanical properties.^12^ Thus, in recent years researchers paid much attention to the non-covalent individualization of SWNTs bundles. The applied dispersants include conjugated polymers, biomolecules, ionic liquids and various surfactants.^13–16^ After noncovalent individualization, SWNTs were sorted by diameter, electronic type, chirality and even handedness to some extent *via* ultracentrifugation,^17^ aqueous two-phase extraction^18,19^ and gel chromatography.^20,21^ Among the dispersants applied, biomolecules are crucial to the medical and diagnostic applications of SWNTs as drug carriers,^8,22^ therapeutic agents^23^ or ultrasensitive biosensors in cellular systems.^24,25^ Because of their biocompatibility, nucleic acids and proteins related biomolecules are initially considered as optimal candidates for the dispersion of SWNTs bundles. The π–π stacking between the heterocyclic nucleobases of single-stranded DNA^17,26,27^ or RNA^28^ and the hydrophobic SWNT surfaces along with the negatively charged phosphate sugar backbone are thought to be responsible for the dispersion of SWNTs bundles in the aqueous medium of nucleic acids.^29^ Moreover, the sequence-dependent interaction between DNA and SWNTs makes it possible to sort DNA-SWNTs in line with their chirality and handedness.^30,31^ Whereas, the effective individual dispersion of SWNTs bundles with proteins and their hydrolysates *i.e.* peptide and amino acids are very limited, even though the interaction between proteins or peptides and SWNTs were extensively investigated.^30–34^

Up to the present, individual dispersion and isolation of commercially available SWNTs have been carried out largely on HiPco^35^ or CoMoCAT^36^ SWNTs produced by catalytic chemical vapor deposition (CVD) with diameters ranging from 0.7 to 1.2 nm. Meanwhile, arc-discharged SWNTs are produced at a temperature higher than the evaporating point of graphite with diameters ranging from 0.8 to 2.0 nm. In comparison with HiPco and CoMoCAT SWNTs, arc-discharged ones are longer, straighter and with fewer defects tending to form large and closely packed ropes,^37,38^ which make them more difficult to be individually dispersed. To the best of our knowledge, there are still no reports on the selective and individual dispersion of typical arc-discharged SWNTs with biomolecules.

**Scheme 1:**
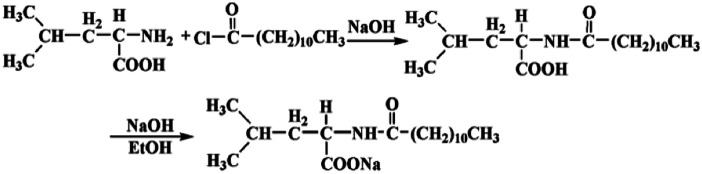
The synthesis procedures of *N*-dodecanoyl leucinate.

Recently, Li *et al.*^39^ reported the dispersion of CoMoCAT SWNTs with the assistance of a commercial available amino acid surfactant *N*-cocoyl sarcosinate, enabling it possible to separate SWNTs in line with diameters by density gradient centrifugation. In this context, the natural α-amino acid surfactant, *N*-dodecanoyl leucinate, was designed and synthesized through the Shortton–Bowman reaction and treatment with sodium hydroxide in alcohol medium (Scheme 1). This natural α-amino acid derivative shows an effective individualization and selective dispersion towards the arc-discharged SWNTs, which is ascribed to the unique molecular structure of *N*-dodecanoyl leucinate inducing charge transfer to SWNTs. The *N*-dodecanoyl leucinate dispersed arc-discharged SWNTs are superior to the *N*-cocoyl sarcosinate dispersed CoMoCAT tubes for their longer length and better biocompatibility.

## 2 Experimental

### 2.1 The synthesis of *N*-dodecyl leucinate

The *N*-dodecanoyl leucinate was synthesized through the Shortton–Bowman reaction.^40^ When 2.6 g of l-leucine dissolved in a round bottom flask containing 25 mL of NaOH (1 mol L^−1^), the pH value of the solution was adjusted with a small amount of HCl (1 mol L^−1^) to 9. After 4.6 mL of *N*-dodecanoyl chloride dropwise added in, the flask was put in an ice bath and kept stirring for 3 hours. The reacting solution was further adjusted with a small amount of HCl (1 mol L^−1^) till its pH value about 6, and then transferred to a 100 mL separatory funnel. After successive extraction with 20 mL of *n*-hexane for three times, the lower aqueous phase was collected and evaporated with a rotatory evaporator to obtain a solid mixture of *N*-dodecanoyl leucine and NaCl. The solid mixture was subjected to Soxhlet extraction with ethanol, and then achieved 1.36 g of *N*-dodecanoyl–l-leucine after evaporation of the solvent with a rotatory evaporator.

The above *N*-dodecanoyl–l-leucine reacted with 0.18 g NaOH in 10 mL of ethanol for 30 minutes, and then 1.5 g of sodium *N*-dodecanoyl leucinate was obtained by filtration and vacuum dying.

### 2.2 Preparation and primary purification of raw SWNTs

The original single-walled carbon nanotubes were prepared by an improved arc discharge procedure.^41^ The anode is Ø6 × 120 mm graphite rod with a drilled Ø4 × 100 mm hole (Ø stands for the diameter of the rod or the hole), which was filled with a powdered mixture of graphite, YNi_2_ alloy and FeS. The cathode is a circular graphite block with a radius of 20 mm. An arc was generated at a current of 100 A under a 500 torr helium atmosphere. After cooling down, the crude SWNTs were collected from the arc discharge chamber.

The crude SWNTs were heat-treated in air at 673 K for 3 hours, and then immersed in concentrated hydrochloric acid for 24 hours to remove the metal catalyst. After washing with large amount of deionized water and drying under vacuum, primarily purified SWNT was obtained.

### 2.3 Dispersion of the primarily purified SWNTs

In a typical dispersion experiment, 10 mg primarily purified single-walled carbon nanotubes were ultrasonically (Sonics, VCX 750, Vibra-cell, USA) dispersed in 100 mL aqueous solution of sodium *N*-dodecanoyl leucinate (3.75 mg ml^−1^) for 12 h at a temperature of 10 °C. The resulting dispersion was then centrifuged with an angle rotor (P70AT2, CP70MX, Hitachi Koki Co.) at a rotation speed of 15 000 rpm for 2 h, and the above 80% supernatant carefully collected, and named as the sodium *N*-dodecanoyl leucinate dispersed SWNTs.

### 2.4 Characterization methods

#### 2.4.1 Optical absorption spectroscopy

The visible near infrared (vis-NIR) optical adsorption spectra were recorded by a computer manipulated dual-beam spectrometer (UV3600, Shimadzu) with a 10 mm quartz cell and a spectral resolution of 0.2 nm.

#### 2.4.2 Atomic force microscope (AFM) observation

AFM observation was performed on an AFM/SPM system (MultiMode8, Bruker). Samples were prepared by casting a drop of the sodium *N*-dodecanoyl leucinate dispersed SWNTs on a clean silicon substrate and drying under vacuum, and then subjected to observation in tap mode.

#### 2.4.3 Transmission electron microscope (TEM) observation

TEM observation was conducted with a transmission electron microscope (JEM-2100, JEOL, Tokyo, Japan) at an accelerator voltage of 200 kV. Samples were prepared by depositing a drop of the sodium *N*-dodecanoyl leucinate dispersed SWNTs on a 200 mesh Cu grid and drying under vacuum.

#### 2.4.4 Raman scattering spectra

Under excitation at 633 nm, a Raman spectrum at room temperature was recorded using a micro laser Raman spectrometer (HR800, Jobin Yvon). Samples were prepared by filtering *N*-dodecanoyl leucinate-dispersed SWNTs with a 0.1 mm polytetrafluoroethylene (PTFE) micromembrane, and then dried under vacuum at 100 °C for 1 day. A 100× air objective is used, and the diameter of the laser spot is approximately 1 mm. The laser power is carefully controlled to avoid any thermal effects on the Raman shift.

#### 2.4.5 Fourier transform-infrared (FT-IR) spectroscopy

FT-IR spectra were recorded on a Fourier transform infrared spectrometer (Spectrum one, PerkinElmer). A solid sample was obtained by filtering the sodium *N*-dodecanoyl leucinate dispersed and drying under vacuum at 100 °C for 24 hours. After grinding with KBr particles and pressing into a module, the samples were measured in transmittance mode.

## 3 Results and discussion

### 3.1 The individualization of SWNTs by *N*-dodecyl leucinate

**Fig. 1:**
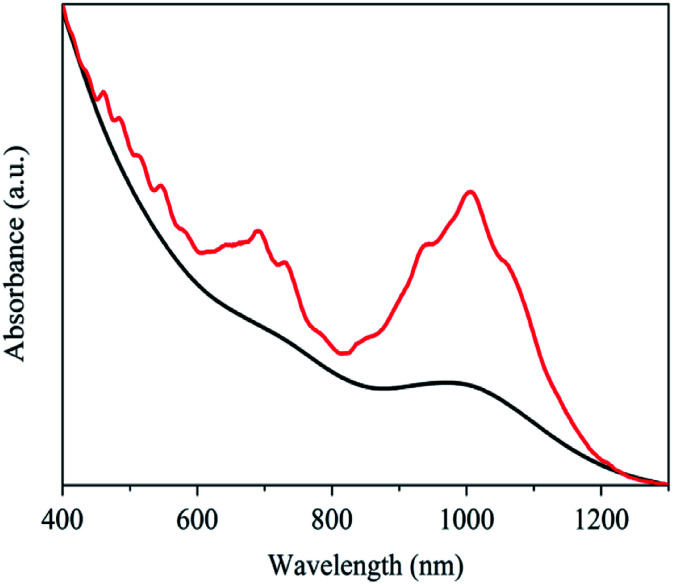
The vis-NIR spectra of the primarily purified (red curve) and the sodium *N*-dodecanoyl leucinate dispersed (black curve) SWNTs.

Optical absorption spectroscopy is a convenient and effective technique to evaluate the aggregating state and to assign the chiral indices (m, n) of SWNTs.^42^ Shown in Fig. 1 are the vis-NIR spectra of the primarily purified and the *N*-dodecanoyl leucinate dispersed SWNTs. In comparison with that of the primarily purified ones, the *N*-dodecanoyl leucinate dispersed SWNTs exhibit significantly enhanced effective absorption and many resolvable fine absorption features, which is a direct result of the individually dispersed SWNTs. Because of the inversely proportional relationship between the nanotube diameter and the energy of the absorption features, the absorption spectrum of individual arc-discharged SWNTs is not as well-resolved as that of individual HiPco or CoMoCAT tubes due to its larger mean tube diameter (∼1.4 nm). In line with the electronic band theory,^43^ the spectral features could be assigned to SWNTs with varying chiral indices (see Fig. S1 and Table S1, ESI†).

**Fig. 2:**
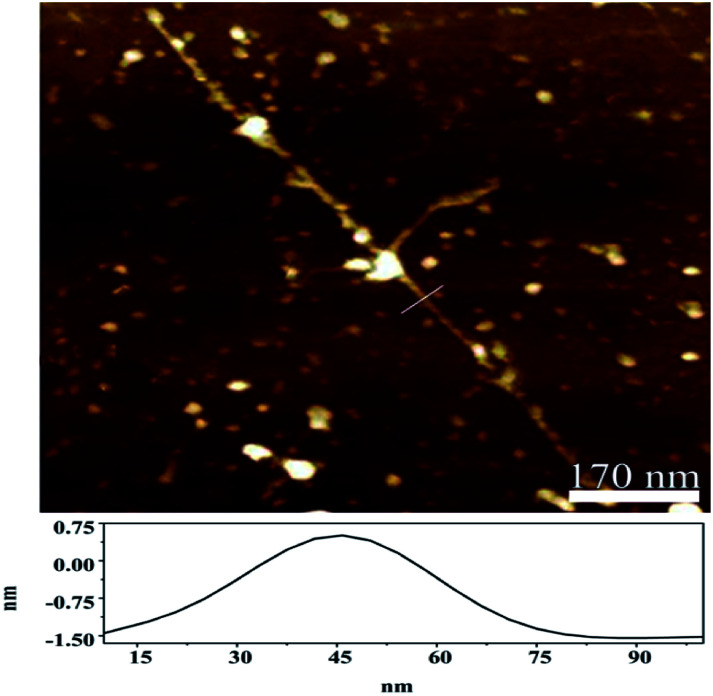
The typical AFM image of the sodium *N*-dodecanoyl leucinate dispersed SWNTs.

Further support for the individualization of arc-discharged SWNTs comes from AFM characterization. Shown in Fig. 2 is a typical AFM image of the *N*-dodecanoyl leucinate dispersed SWNTs, in which individually dispersed SWNTs with rough surfaces are observed. The white dots observed on the substrate are considered to be the molecular aggregates of sodium *N*-dodecanoyl leucinates. It evidences that SWNTs are well dispersed and coated by *N*-dodecanoyl leucinate with varying thickness. Height analysis (indicated by the white solid line in the image) produced a value of 1.8 nm. Considering the varying orientation of the dodecanoyl and the varying thickness of the coating on SWNTs, it is reasonable for us to assign the SWNTs observed here as individual. Moreover, it can be estimated from Fig. 2 that the length of the nanotube is about 1 μm, which is much longer than those individually dispersed HiPco^38^ or CoMoCAT^44^ SWNTs. For comparison, the AFM observation of the primarily purified SWNTs was also performed with a sample prepared by supersonic dispersion and centrifugation of the primarily purified SWNT in dimethylformamide. It can be seen from Fig. S2a (ESI†) that there are many carbon nanoparticles aggregated around SWNTs bundles, and the tubes localized only at isolated regions of the substrate. The high-level analysis (represented by the solid yellow line in the Fig. S2b, ESI†) yielded a value of 5.8 nm, which is much higher than that of the sodium *N*-dodecanoyl leucinate dispersed SWNTs.

**Fig. 3:**
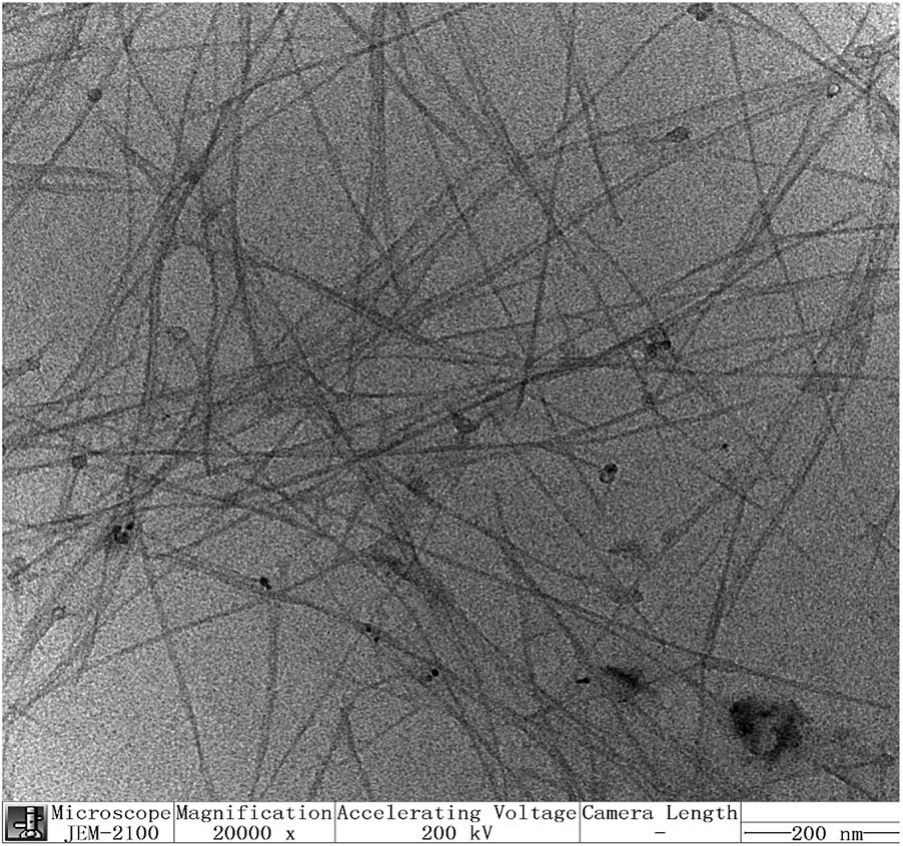
The typical TEM image of the sodium *N*-dodecanoyl leucinate dispersed SWNTs.

Shown in Fig. 3 is the typical TEM image of the sodium *N*-dodecanoyl leucinate dispersed SWNTs. It is obvious that most of the sodium *N*-dodecanoyl leucinate dispersed SWNTs are relatively straight with an average length about 1000 nm, and they are quite uniformly isolated without specially entangled or agglomerated areas. Moreover, the sodium *N*-dodecanoyl leucinate dispersed SWNTs are observed as thin bundles with an average diameter around 10 nm, which is smaller than that of bundles prepared by solubilisation of SWNTs in solution of low molecular weight surfactants.^45^ The thin bundles of SWNTs are assumed to be formed from their corresponding individual ones in water media during the preparation of TEM samples. Thus, the TEM image offers another support for the good ones in water media during the preparation of TEM samples. Thus, the TEM image offers another support for the good dispersing ability of sodium *N*-dodecanoyl leucinate towards the arc-discharged SWNTs.

**Fig. 4:**
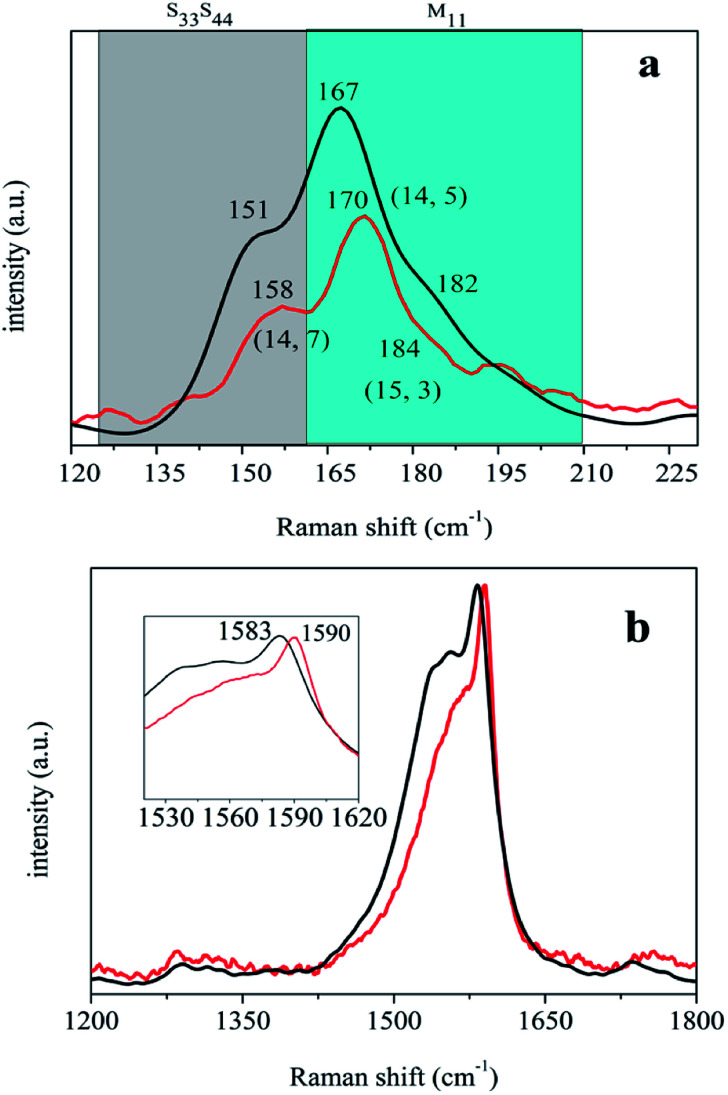
The normalized Raman spectra of the primarily purified (black curve) and the sodium *N*-dodecanoyl leucinate dispersed (red curve) SWNTs in the ranges of 120–230 cm^−1^ (a) and 1200–1800 cm^−1^ (b), respectively.

Resonance Raman spectroscopy is a powerful technique to characterize carbon materials. Particularly, the weak RBM band and the moderate split G band are fingerprints of SWNTs. To investigate further the special dispersing ability of *N*-dodecanoyl leucinate towards arc-discharged SWNTs, resonant Raman spectroscopy was performed under excitation at 633 nm, which will bring varying metallic and semiconducting arc-discharged SWNTs into resonant scattering. Showed in Fig. 4 are the normalized Raman spectra of the primarily purified and the *N*-dodecanoyl leucinate dispersed SWNTs. As shown in Fig. 4a, the Raman RBM peaks of the *N*-dodecanoyl leucinate dispersed SWNTs are systemically blue-shifted in comparison with those of the primarily purified SWNTs. A similar blue-shift was reported previously for soluble SWNTs under varying laser excitations.^46^ According to O'Connell *et al.*^47^ the apparent blue-shift in RBM frequency was a direct result of bundling-induced red-shifts of the electronic transition, which makes it possible for SWNTs with different diameter in bundle or in individual states to resonate with the same laser excitation. Owing to the curvature of the rolled-up graphite sheet^48^ G band splits to a few sub-groups, *e.g.* the lower frequency component (G^−^) associated with vibrations along the circumferential direction and the higher frequency component (G^+^) attributed to vibrations along the direction of the nanotube axis. It is observed from Fig. 4b that the upper sharp G^+^ band is also blue-shifted from 1583 cm^−1^ to 1590 cm^−1^ when the primarily purified SWNTs are dispersed by *N*-dodecanoyl leucinate. We believe that the release of tube–tube interaction and the wrapping of SWNTs by *N*-dodecanoyl leucinate anions might mainly be responsible for such blue-shift.

The Radial breathing mode (RBM) peaks in Raman spectra are assignable to SWNTs species according to the referencing Kataura plot and the wavelength of the excitation laser. In 633 nm excitation spectrum, the bands between 160 to 210 cm^−1^ range are ascribed to metallic SWNTs, and the strong peak at 170 cm^−1^ and the shoulder at 184 cm^−1^ observed in Fig. 4a could be assigned to the metallic carbon nanotube with indices of (14,5) and (15,3). Whereas, the bands between 125 to 160 cm^−1^ range are due to semiconducting SWNTs, and the moderate peak at 158 cm^−1^ could be assigned to the semiconducting carbon nanotube with index of (14,7). It is estimated from Fig. 4a that the relative intensity of semiconducting SWNTs to metallic ones is a little elevated after the primarily purified SWNTs are dispersed by *N*-dodecanoyl leucinate. Moreover, as shown in Fig. 4b the asymmetric Breit–Wigner-Fano satellite G^−^ band for *N*-dodecanoyl leucinate dispersed SWNTs gets narrower and weaker than that for the primarily purified, indicative of the decrease in metallic SWNTs. Thus, it is concluded that *N*-dodecanoyl leucinate has selective dispersing ability towards semiconducting SWNTs.

### 3.2 Charge transfer from *N*-dodecanoyl leucinate to SWNTs

**Fig. 5:**
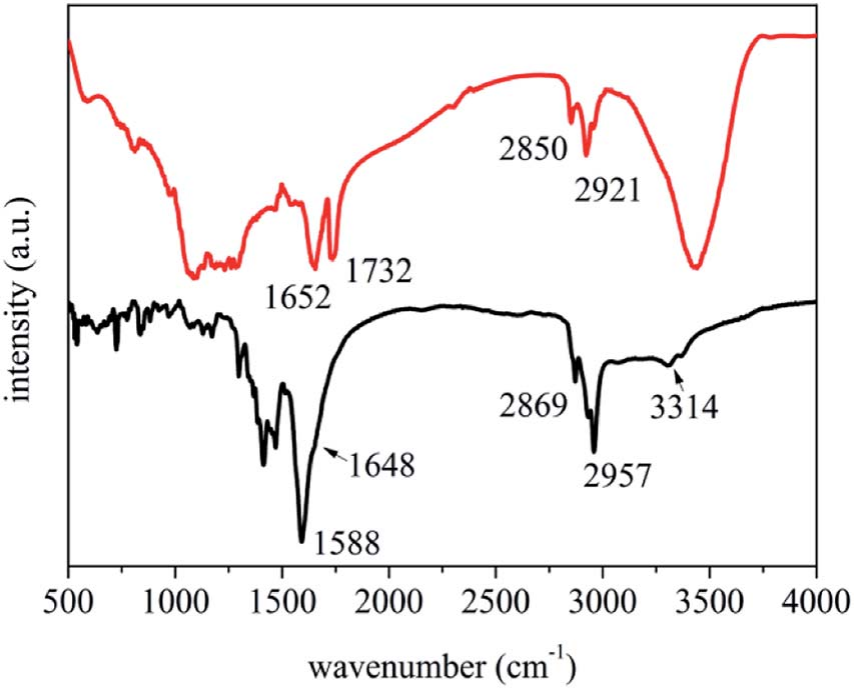
FT-IR spectra of *N*-dodecanoyl leucinate (black curve) and the *N*-dodecanoyl leucinate dispersed SWNTs (red curve).

As a matter of fact, the adsorption of *N*-dodecanoyl leucinates on SWNTS could be regarded as a charge transfer process. Shown in Fig. 5 are the FT-IR spectra of the sodium *N*-dodecanoyl leucinate and the sodium *N*-dodecanoyl leucinate dispersed SWNTs. In comparison with those observed in *N*-dodecanoyl leucinate, the amide group related vibration bands at 1588 cm^−1^ (C=O stretching vibration), 1648 cm^−1^ (*N*–H bending vibration) and 3314 cm^−1^ (*N*–H stretching vibration) are systemically blue-shifted. The strengthening of amide bonds is a result of the charge transfer from the partial occupation of the low lying antibonding donator orbital of *N*-dodecanoyl leucinate to SWNTs. Additionally, it can also be seen that the C–H stretching vibrations in 2800–3100 cm^−1^ of CH_2_ and CH_3_ are systemically red-shifted, indicating the existence of hydrophobic CH–π interactions between the linear *N*-dodecyl of *N*-dodecanoyl leucinate and the curved graphene surface of SWNTs. It is estimated that there is net electric charge transferred from *N*-dodecanoyl leucinate to SWNTs, because of the strong electron-donating ability of amides.

According to Wise *et al.*,^49^ donating charge from SWNTs would result in a blue-shift while accepting charge to SWNTs results in a red-shift in the G^+^ band around 1592 cm^−1^. Monteiro *et al.*^50^ and Salvatierra *et al.*^51^ also correlated the shift of Raman G band with the transfer of charge for the individually dispersed SWNTs. It can be seen from the inset of Fig. 4b that the G^+^ band is blue-shifted from 1583 cm^−1^ to 1590 cm^−1^ when the primarily purified SWNTs are dispersed by *N*-dodecanoyl leucinate. In contrast, the G^+^ band is blue-shifted to 1596 and 1601 cm^−1^ when the primarily purified SWNTs are dispersed by DOC and polymethyl(1-undecylic acidyl)silane, respectively.^46^ However, it should be noted that the electron-accepting polymethyl(1-undecylic acidyl)silane, having selective dispersing ability towards metallic SWNTs, leads to the largest shift in G^+^ band. Whereas, the electron-donating *N*-dodecanoyl leucinate, having selective dispersing ability towards semiconducting SWNTs, induces to the smallest shift in G^+^ band. In this context, we correlate the shift in G^+^ band with the charge transfer between surfactants and SWNTs and the selective dispersion of surfactants towards SWNTs with varying conductivity. Therefore, the selective dispersion of semiconducting SWNTs by *N*-dodecanoyl leucinate is a result of the charge transfer from *N*-dodecanoyl leucinate to SWNTs.

### 3.3 Interactions between SWNTs and *N*-dodecanoyl leucinate

In order to explain the good recognition of SWNTs by amino acid surfactant *N*-cocoyl sarcosinate, Li *et al.*^39^ proposed the essential π–π stacking interaction between the planar conjugated amide group of *N*-cocoyl sarcosinate and the π electrons of SWNTs. The amide groups of amino acid surfactants were anchored on SWNTs by π–π stacking effects, and then the alkyl chains closely assembled around SWNTs in line with their chiral angles by hydrophobic interactions. Analogously, the good dispersing ability of *N*-dodecanoyl leucinate towards arc-discharged SWNTs could also be ascribed to the important π–π stacking effect. Nonetheless, the *N*-dodecanoyl leucinate is superior to *N*-cocoyl sarcosinate in the dispersion of SWNTs. Though both of them have a linear alkyl chain, their planar-conjugated amide groups as well as the alkyls linking the amide and carboxyl groups are different (see Fig. S3, ESI†). Substitution of the methyl in the amide group of *N*-cocoyl sarcosinate with hydrogen atom in *N*-dodecanoyl leucinate is beneficial for the formation of π–π stacking interactions owing to the decrease in steric hindrance. Furthermore, the methylene linking the amide and carboxyl groups in *N*-cocoyl sarcosinate is attached with an isobutyl side chain in *N*-dodecanoyl leucinate. The side chain offers additional hydrophobic interaction with SWNTs and larger buffing effect to the hydrophilic pull force from carboxyl groups, which is also beneficial for the π–π stacking interactions between amide groups and SWNTs.

Apart from the above discussed hydrophobic, hydrophilic and π–π tacking effects. The dispersion of SWNTs with surfactants was also accounted for by electrostatic interactions. The negatively charged *N*-dodecanoyl leucinate anchoring on SWNTs provide repulsive force for nanotube dispersion, and the sodium ions among individual SWNTs also play an important role in the dispersion of SWNTS in the aqueous solution of sodium *N*-dodecanoyl leucinate. In sharp contrast to sodium *N*-dodecanoyl leucinate, both leucine and *N*-dodecanoyl leucine show very poor dispersing ability towards arc-discharged SWNTs (see Fig. S4, ESI†), confirming the essential effect of electrostatic interactions.

### 3.4 Potentials of the *N*-dodecanoyl leucinate dispersed SWNTs

On one hand, the sodium *N*-dodecanoyl leucinate dispersed SWNTs are the first natural α-amino acid derivative dispersed SWNTs. They are expected to be biocompatible and will find applications in the field of medical diagnostic and therapeutic agents as biosensor, drug delivery, and enzyme supporter, *etc.*^52^ On the other hand, the individually dispersed SWNTs by sodium *N*-dodecanoyl leucinate are applicable in the aspect of fundamental studies of SWNTs. The effective individualization of SWNTs with sodium *N*-dodecanoyl leucinate makes it possible to separate arc-discharged SWNTs by diameter, electronic type, chirality, and even handedness (optical isomer) by several postsynthetic separation techniques. The effective individualization of SWNTs with sodium *N*-dodecanoyl leucinate also enables us to measure the extinction coefficients of arc-discharged SWNTs at S_11_, S_22_, M_11_ and S_33_ absorption bands, respectively (see Fig.s S5–S7, Table S2, ESI†).

The outstanding advantage of the noncovalent amino acid surfactant individualization of SWNTs is that their intrinsic electronic and mechanical properties are preserved. Therefore, it might not be necessary to get rid of surfactants in some application like chemical or biological sensor and miniaturization of electronic circuitry, *etc.* Nonetheless, the surfactant *N*-dodecanoyl leucinate could be gotten rid of by acid or base hydrolysis. *N*-dodecanoyl leucinate is anchored on SWNTs mainly by π–π stacking effects between the amide groups in amino acid surfactant and the graphene wall of SWNTs. The amide bond in *N*-dodecanoyl leucinate could be broken by refluxing the sodium *N*-dodecanoyl leucinate dispersed SWNTs in 6 mol L^−1^ HCl or 5 mol L^−1^ NaOH solutions. With the breakage of amide bond, hydrolysis products, leucine (or leucinate) and *N*'dodecanoyl carboxylic acid (or *N*-dodecanoyl carboxylate), could be washed out with large amounts of organic or inorganic solvents. Alternatively, since the amino acid surfactant is physically adsorbed on the surfaces of SWNTs, *N*-dodecanoyl leucinate could also be gotten rid of by heat treatment at a temperature around 200 °C under an inert atmosphere.

## 4 Conclusions

Arc-discharged SWNTs were effectively and selectively dispersed by *N*-dodecanoyl leucinate, which could be elucidated by the charge transfer from *N*-dodecyl leucinate to SWNTs. It is expected that the biocompatible natural α-amino acid derivative dispersed SWNTs will found applications in the field of medical diagnostic and therapeutic agents.

## Conflicts of interest

There are no conflicts to declare.

## Supplementary Material

RA-010-D0RA02862B-s001
