# Supplementary material for: Dispersion of arc-discharged single-walled carbon nanotubes using the natural α-amino acid derivative N-dodecanoyl leucinate
Source: RSC Adv. 2020 Jun 5;10(36):21643–9. doi: 10.1039/d0ra02862b (PMC9054530; doi:10.1039/d0ra02862b)
Supplement: RA-010-D0RA02862B-s001 [file RA-010-D0RA02862B-s001.pdf]

Electronic Supplementary Material (ESI) for RSC Advances.
This journal is © The Royal Society of Chemistry 2020

Dispersion of arc-discharged single-walled carbon nanotubes using the natural α-amino acid derivate *N*-dodecanoyl leucinate

Heng Zhao,[a] Lihua Guo*[b] and Yongfu Lian *[a]

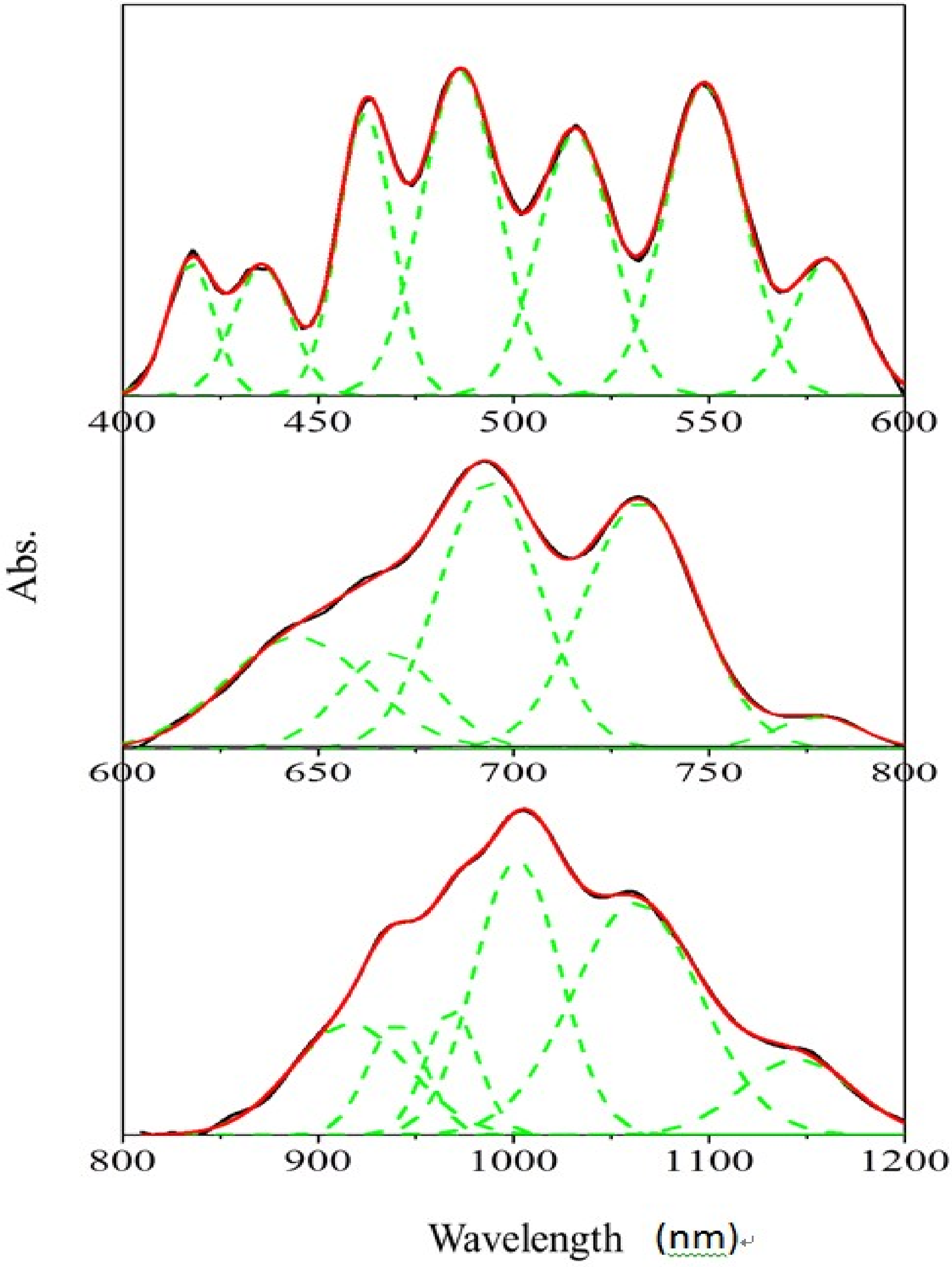

Figure S1 Gaussian fitting of the optical absorption spectrum of the sodium *N*-dodecanoyl leucinate dispersed SWNTs.

Table S1 Assignment of the optical absorption features to the interband transions and chiral indices of SWNTs.

Absorption peaks （nm）	Interband transitions	Calculated diameters (nm)	Chiral indices （n， m）
416.3	S_{44}	1.406	（16,3）
436.2	S_{33}	1.175	（11,6）
461.8	$2S_{33}$	1.244	(14,3)
486.3	$3S_{33}$	1.311	(10,9)
518.5	S_{33}	1.397	（14,6）
548.0	S_{33}	1.477	（14,7）
585.0	S_{33}	1.575	（12,11）
732.9	M_{11}	1.482	（15,6）
777.1	M_{11}	1.571	（13,10）
673.2	M_{11}	1.361	（10,10）
641.8	M_{11}	1.298	（11,8）
695.2	M_{11}	1.404	（13,7）
942.8	S_{22}	1.270	（13,5）
1010.4	S_{22}	1.361	（16,2）
1054.9	S_{22}	1.421	（14,6）
905.3	S_{22}	1.220	（14,3）
978.1	S_{22}	1.318	(10,9)

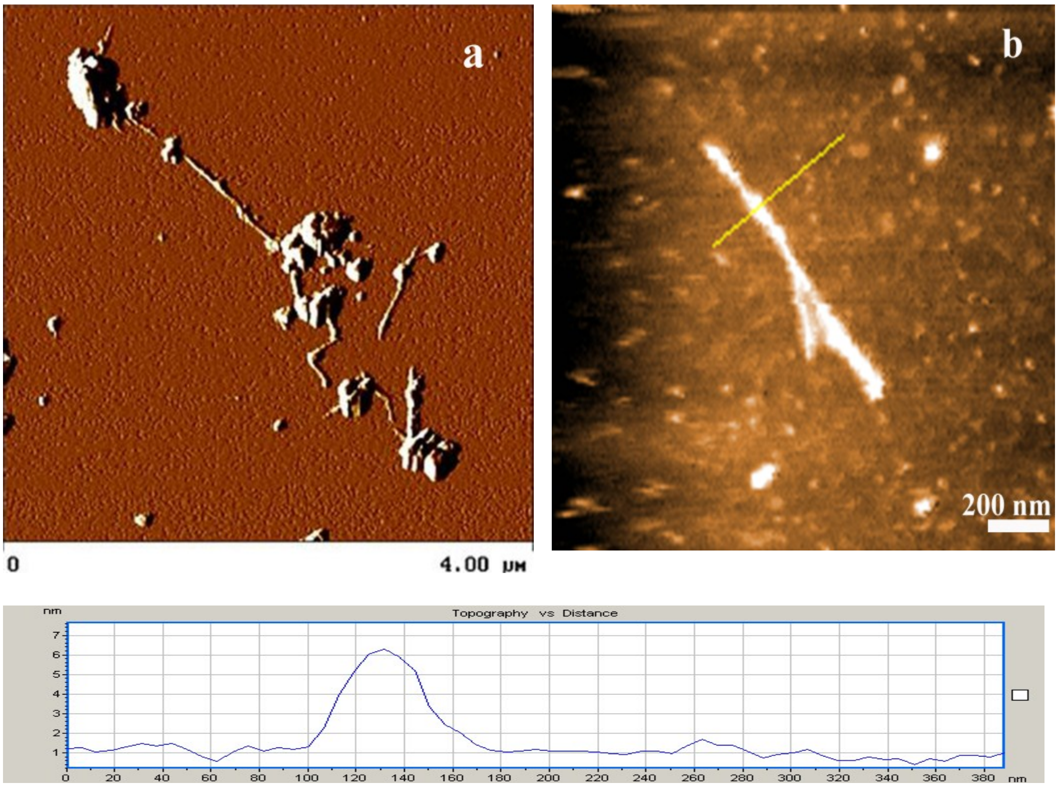

Figure S2. AFM of primary purified single-walled carbon nanotubes.

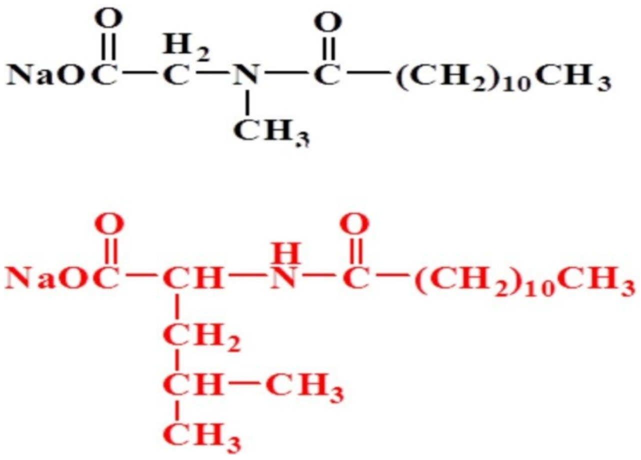

Figure S3 Molecular structures of sodium *N*- cocoyl sarcosinate (black) and sodium *N*-dodecanoyl leucinate (red).

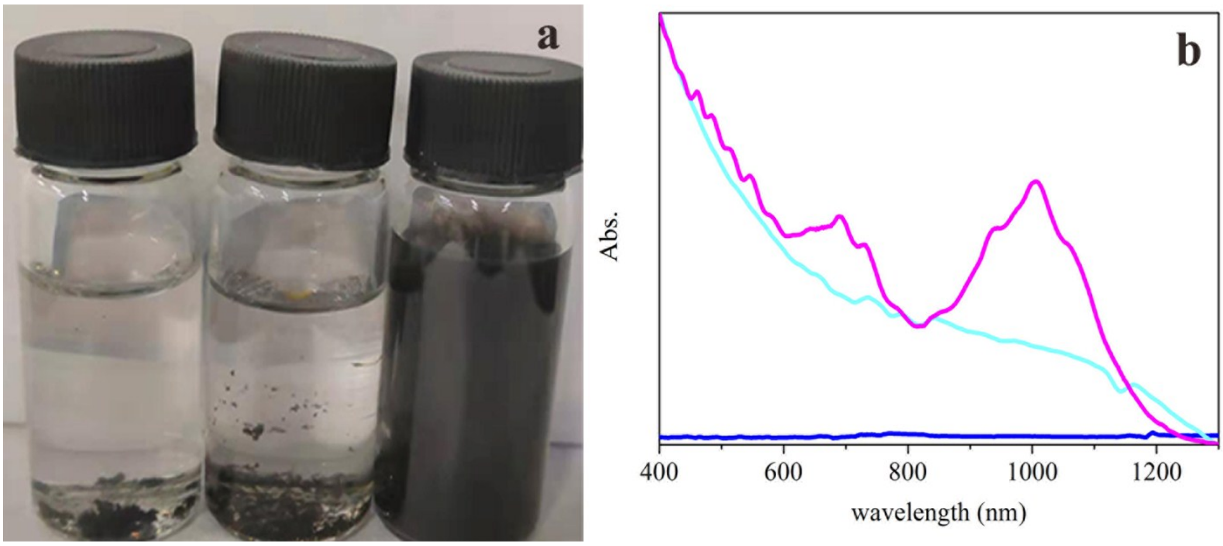

Figure S4 The photos (from left to right) (a) and optical absorption spectra (from bottom to top) (b) of the leucine, *N*-dodecanoyl leucine and sodium *N*-dodecanoyl leucinate dispersed SWNTs, respectively.

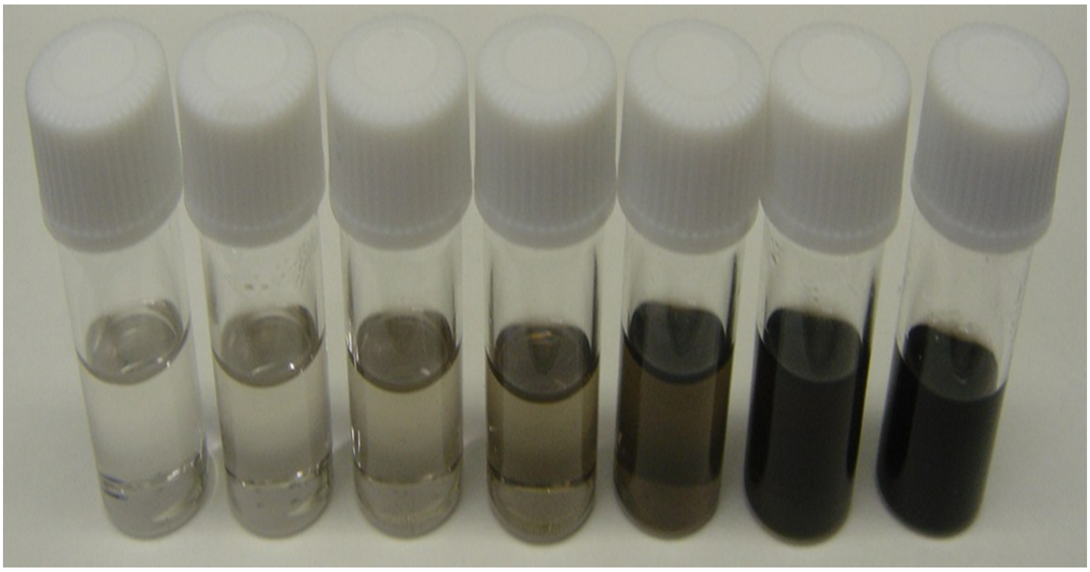

Figure S5 The photos (from left to right) of sodium *N*-dodecanoyl leucinate dispersed SWNTs with concentrations of 1.2, 2.3, 4.7, 9.4, 18.8 and 37.5 μg/ml, respectively.

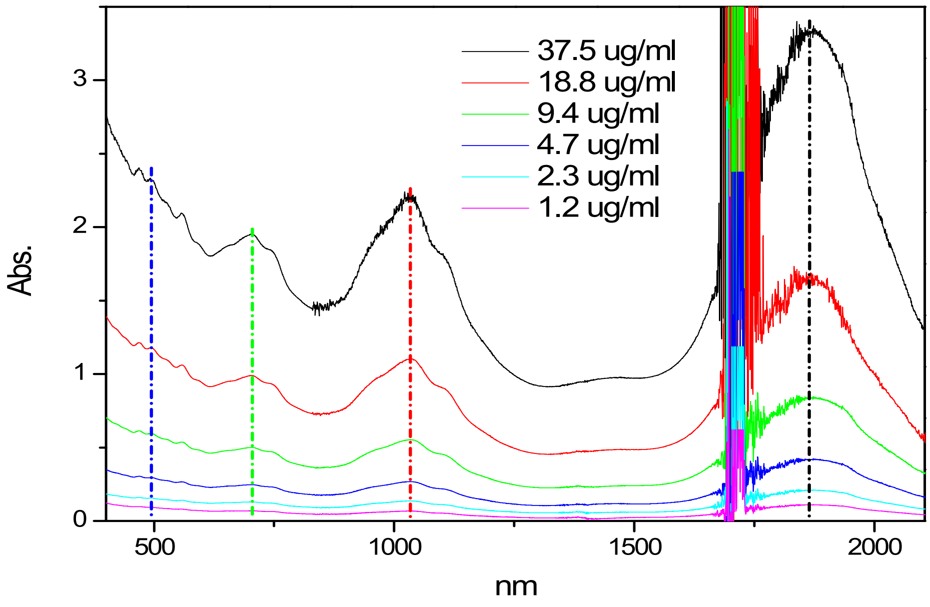

Fig S6 Vis-NIR spectra of the dispersed SWNTs with varying concentrations of sodium *N*-dodecanoyl leucinate

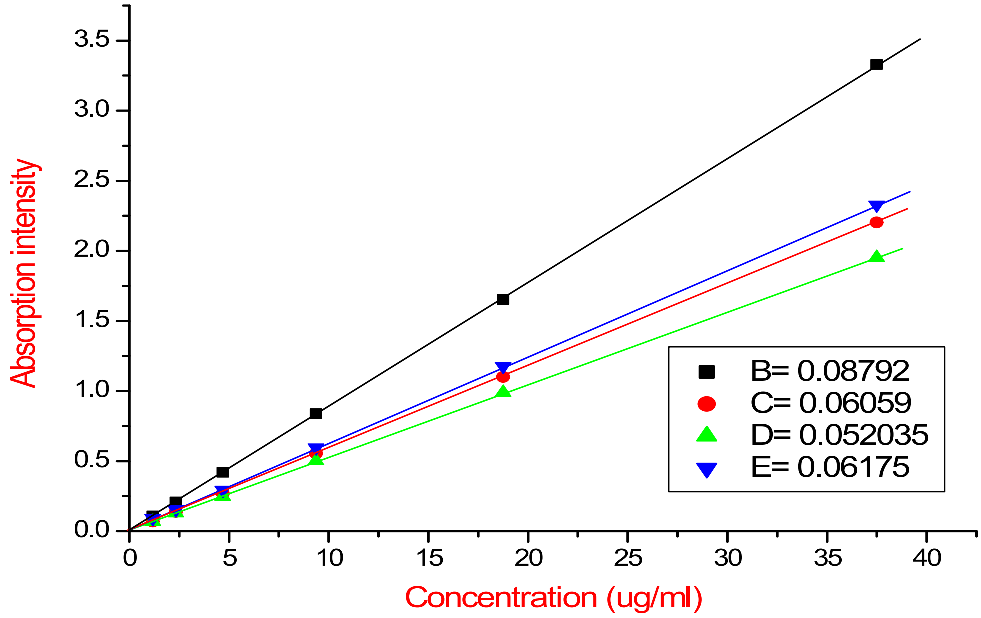

Fig S7 The absorbance dependence on the concentration of the sodium *N*-dodecanoyl leucinate dispersed SWNTs at S_{33} (blue) S_{22} (red) M_{11} (green) and S_{11} absorption bands, respectively.

Table S2 The extinction coefficients of the sodium *N*-dodecanoyl leucinate dispersed SWNTs at S_{33}, S_{22}, M_{11} and S_{33} absorption bands, respectively.

	S_{33} (493 nm)	M_{11} (700 nm)	S_{22} (1037 nm)	S_{11} (1850 nm)
Slope	0.06175	0.05204	0.06059	0.08792
ε(mg/mL)$^{-1}$ cm^{-1}	61.75	52.04	60.59	87.92
